# The Allen Ancient DNA Resource (AADR) a curated compendium of ancient human genomes

**DOI:** 10.1038/s41597-024-03031-7

**Published:** 2024-02-10

**Authors:** Swapan Mallick, Adam Micco, Matthew Mah, Harald Ringbauer, Iosif Lazaridis, Iñigo Olalde, Nick Patterson, David Reich

**Affiliations:** 1 Department of Genetics, Harvard Medical School, Boston, MA 02115 USA; 2 Broad Institute of MIT and Harvard, Cambridge, MA 02142 USA; 3 Howard Hughes Medical Institute, Boston, MA 02115 USA; 4 Department of Human Evolutionary Biology, Harvard University, Cambridge, MA 02138 USA; 5 Max Planck Institute for Evolutionary Anthropology, Leipzig, 04103 Germany; 6 BIOMICs Research Group, University of the Basque Country, 01006 Vitoria-Gasteiz, Spain

**Keywords:** Genetic variation, Genetic variation

## Abstract

More than two hundred papers have reported genome-wide data from ancient humans. While the raw data for the vast majority are fully publicly available testifying to the commitment of the paleogenomics community to open data, formats for both raw data and meta-data differ. There is thus a need for uniform curation and a centralized, version-controlled compendium that researchers can download, analyze, and reference. Since 2019, we have been maintaining the Allen Ancient DNA Resource (AADR), which aims to provide an up-to-date, curated version of the world’s published ancient human DNA data, represented at more than a million single nucleotide polymorphisms (SNPs) at which almost all ancient individuals have been assayed. The AADR has gone through six public releases at the time of writing and review of this manuscript, and crossed the threshold of >10,000 individuals with published genome-wide ancient DNA data at the end of 2022. This note is intended as a citable descriptor of the AADR.

## Background & Summary

**Fig. 1:**
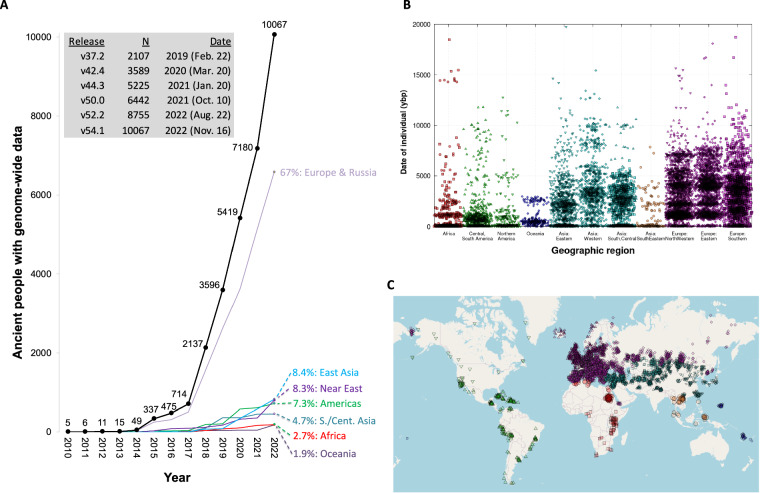
Growth in world’s published human genome-wide ancient DNA data. (**A**) By year of publication (broken down by geography). (**B**) By date (color and symbol both indicate geographic location). (**C**) By geography (using same color and symbol scheme as in previous panel).

The first genome-wide ancient DNA data were published in 2010^1–3^. However, it was only in 2015 with the advent of large-scale studies of Holocene genomes, in-solution enrichment of ancient DNA libraries for targeted single nucleotide polymorphisms (SNPs)^4–6^, and the introduction of automated protocols and liquid handling robots for processing of ancient DNA libraries^7,8^, that the number of individuals with genome-wide data began to increase rapidly. Between 2010 and 2014, data from an average of about 10 individuals with genome-wide data were published each year. Between 2015 and 2017, the numbers increased to about 200 annually. Since 2018, data from thousands of individuals have been published every year (Fig. 1). About 67% of the data are from Europe and Russia, a proportion that has held relatively steady since the beginning of the field of ancient DNA. The proportion of data from East Asia has grown from about 1% of all data in 2015 to 8% today. The proportion of data from Africa has grown from none in 2014 to 3% today (Fig. 1).

A challenge in analyzing ancient DNA data is that it has been reported over hundreds of independent studies. Thus, while raw sequence data for more than 99% of individuals^9^ are fully available in public repositories such as ENA^10^ and SRA^11^, the uploaded data exist in diverse formats, as do the meta-data such as archaeological, chronological, and geographic information. Some resources exist which consolidate subsets of publicly available ancient DNA data, including a Y-chromosome database with assembled information from nearly two thousand ancient Eurasian individuals^12^, a mitochondrial DNA database with more than two thousand individuals^13^, and the Online Ancient Genome Repository^14^ which copies publicly available data and encapsulates each dataset into an archived tar file. However, none of these provide a regularly curated dataset that attempts to include all published data in an easily co-analyzable format, such as a single genotype file with complete annotations in a single tab-delimited form.

### Sources of data

To bring data generated outside our own laboratory into the AADR, we usually start with available sequences from a public repository, most often the European Nucleotide Archive (https://www.ebi.ac.uk/ena), following accession numbers given in the published papers. In some cases we start with alternatively formatted versions that we request directly from the authors. All source articles are cited in the reference list of this paper. For data generated in our laboratory, we start from our own raw sequence files, which are the basis for data uploaded to established public repositories.

## Methods

The raw data generated outside our laboratory come in diverse formats, usually *fastq* files (for raw sequence data) or *bams* (for either unaligned reads or reads aligned to a reference genome)^15^. A challenge is that there can be considerable variation in *fastq* and *bam* files, reflecting the formatting, filtering and processing choices made by researchers in generating data. This includes:

- Base calls and associated quality scores in raw sequences are often modified by the researchers who generated the data. One common modification is to recalibrate base quality scores^16^. Another modification is to ignore information from the ends of sequences, either by masking terminal bases in the sequences that are uploaded and marking them as “N”, or clipping (removing) them altogether^17^. This reduces error rates associated with cytosine deamination typical of ancient DNA data. However, it also means that users cannot make choices about whether to use the valuable data that have been masked and clipped (such as sites unaffected by deamination). In addition, this procedure has the effect of making it difficult to identify damaged molecules which are a strong indicator that those molecules indeed are ancient and not derived from some potential contaminating modern human source.
- Sequences may be aligned to different human reference genomes, typically hg19, hs37d5, or hg20, each with their own unique coordinate systems. To build a homogeneous dataset, we therefore have to map to a unified coordinate system, currently based on hg19^18–20^. A further challenge is that chromosomes may have inconsistent naming conventions (for example ‘chr1’ v. ‘1’, or ‘chrMT’ v. ‘MT’ v. ‘chrM’), or the sorting order of chromosomes can differ. This results in practical difficulties in merging datasets.
- Data may be deposited either (i) by library, or (ii) by-individual with multiple libraries in a single file. If data are deposited by library, then it may be necessary to identify and perform a merging step. There are pitfalls that arise in such merging, as in some cases “readgroup” names (a tag which groups reads together) are the same across individuals, and so joint processing of many individuals can inadvertently lead to in-silico contamination.

### Filtering of data

To add data to the AADR, we manually process the dataset from each individual, tailoring the processing procedure according to the characteristics of the data. We create a *bam* file aligned to the hg19 genome reference sequence. The bam files used to generate AADR constitute tens of terabytes in size altogether. We process these bams to produce genotypes at a set of about 1.23 million SNPs that have been assayed for nearly all published individuals with ancient DNA data. For the great majority of ancient DNA datasets, the genotypes are “pseudohaploid”, meaning that we represent the individual by a randomly sampled sequence at each analyzed position. For the small fraction of individuals for whom coverage is sufficient to allow full genotyping, we also release diploid genotypes^21,22^.

### Combining datasets

To increase the usefulness of the AADR, we have added into the AADR data from diverse modern humans, including shotgun sequencing data from sets of individuals included within the 1000 Genomes Project^23^, the Simons Genome Diversity Project^24^, and the Human Genome Diversity Project^25^ To integrate these data, we had to address challenges of different reference genomes (for example transforming from hg20 to hg19 coordinates). There are 6399 modern individuals with shotgun data in the v54.1 AADR release.

We also integrated a dataset of 4114 modern individuals genotyped on the Affymetrix Human Origins array at approximately ~600,000 SNPs^26^. This is a sufficiently valuable dataset that the AADR provides two releases: one on all 1.23 million targets (excluding the Human Origins data), and one on just the Human Origins targets.

Since the v52.2 release, we have also maintained a mitochondrial repository, which now includes mitochondrial genomes for 4122 ancient individuals in the AADR.

## Technical Validation

Prior to each release, several steps are performed to verify that new and updated data components have been added correctly and are co-analyzable with the full datasets.

An initial assessment considers coverage of each individual and evidence for contamination, updating annotations as needed. In addition, we manually curate the genotypes to check that the data from each individual has sensible population genetic properties, by looking for potential outliers based on ADMIXTURE^27^ and principal components analysis^28^.

### Curation of metainformation and integration of ongoing community feedback

Because we are trying to keep AADR current, we err on the side of inclusivity, and thus try to bring data into the dataset even when meta-information and metrics are incomplete. Each AADR release updates meta-information and identifiers as appropriate. We rely on ongoing curation of the dataset as well as feedback from the user community which we invite through communication with the corresponding authors, to identify individuals with erroneous meta-information or corrupted genetic data, which we then seek to correct in subsequent releases.

## Data Record

The AADR dataset is available at Harvard Dataverse^29^ (https://dataverse.harvard.edu/dataverse/reich_lab). The latest release at the time of writing and peer review is 8.0.

Each data release consists of three standard files in EIGENSTRAT format (*.ind*,*.snp*, and*.geno*). We also include an annotation file that is rich in meta-information for the dataset (*.anno*). The*.anno* file includes meta-data manually extracted from the papers reporting the data, in some cases supplemented by information that appeared later or that reflect clarifications from authors or the user community. For archaeological information, we attempt to provide:

- Skeletal codes and grave numbers and sometimes other identifiers, always also including the code used for genetic analysis.
- Latitude and longitude.
- Location information, with a separate column for “Political entity” such as country, and locality information.
- Chronological information in a standard format. When a radiocarbon date is available, we include the laboratory number and calibrated 95.4% confidence interval obtained in OxCal v4.4.2 using either the IntCal20 or SHCal20 calibration curve (if we make an alternative choice, it is explicitly explained in a “Methods for Determining Date” column). We also report the posterior mean and standard deviation of the calibrated radiocarbon date. When no radiocarbon date is available, we present a date uncertainty range based on archaeological context, usually rounded to the nearest 50 or 100 years, and quote the mean and standard deviation assuming a uniform distribution over its range (the standard deviation of a uniform distribution is the range of that distribution divided by the square root of 12).
- We include an estimate of the age of the individual at their death based on physical anthropology when we are able to obtain it.
- We include a group name for the individual, using a naming convention that aims to be systematic^30^.
- Data from individuals generated using shotgun sequencing methods have a suffix “.SG” (for pseudohaploid representations) or “.DG” (for diploid representations).
- We include many metrics computed on the genetic data, including not just amount of data (such as average coverage assayed at the subset of 1.15 million autosomal sites targeted in the 1.23 million SNP enrichment assays), but also molecular sex determination, cytosine-to-thymine rate in the final nucleotide^31^, fraction of the genome in multi-megabase runs of homozygosity^32^, identification of close relatives in the dataset (in a dedicated “family information” column), and estimates of contamination^33,34^. We have added additional metrics in each release to further improve the usefulness of the dataset.
- When data from an individual have been published in multiple studies using the same methodology such as in-solution enrichment, the AADR typically includes only the best quality version which is usually the latest one (for such individuals, the “publication” columns in the*.anno* file notes the date of the publication that first reported data from the individual, as well as the publication that report the version that is actually included within the AADR). For some individuals, we include multiple representations of data, for example from shotgun sequencing, in-solution enrichment, restricted to UDG-treated libraries, or restricted to sequences showing characteristic ancient DNA damage to reduce the possible impact of contaminating sequences (“_d” suffix). The different versions have unique “Version IDs” but the same “Master ID” (which seeks to uniquely identifies an individual). These IDs may change from data release to data release; if data from two different Master IDs are found to come from the same individual, they are merged into a single Master ID.
- The group name may include a suffix that mark individuals such as potentially contaminated (“_contam”), or as a population genetic outlier (“_o”), or as having relatively little data (low coverage – “lc”).

### Citation guidance

Researchers who use the AADR as the starting point for analyses are encouraged to give two citations for the individual datasets: (1) this paper, and (2) the AADR Dataverse citation^29^ specifying the version of the AADR downloaded. Citing the AADR paper is not a substitute for citing the original publications that produced data, which should be specifically referenced in each publication. Supplementary Data Table 1 provides the full list of references in the component papers for the v54.1 release, and will be updated at Dataverse for each new release going forward^29^.

All source articles are additionally cited in the reference list of this paper^1–6,8,25,26,35–260^.

### Supplementary information

Table S1

## Data Availability

The pipeline used for processing raw data generated within the Reich lab is available in the ‘Workflow Description Language’ (WDL) here: https://github.com/DReichLab/adna-workflow, and includes individual python scripts for components of the pipeline.
